# Histological surprise of parotid tuberculosis: about two cases

**DOI:** 10.11604/pamj.2019.32.85.17173

**Published:** 2019-02-20

**Authors:** Hamza Belatik, Lotfi Aouinti, Nabil Touiheme, Hicham Attifi, Karim Nadour, Ali El Boukhari

**Affiliations:** 1 Department of Otolaryngology and Cervico-Facial Surgery, Moulay Ismail Military Hospital, Meknès, Morocco

**Keywords:** Parotid, tuberculosis, parotidectomy

## Abstract

Parotid tuberculosis remains a very rare localization in the Department of Otolaryngology and Cervico-Facial Surgery (ENT) sphere. It is presented in the form of a deceptive clinical picture causing confusion with other pathologies of the parotid gland, including tumor pathology. In addition, its lack of knowledge by practitioners increases the risk of missing the diagnosis. Often, the diagnosis is a histological surprise on a piece of excision after an exploratory parotidectomy. However, its treatment is primarily medical if the positive diagnosis is well established. We report medical observation of two new cases aged 44 and 45 respectively, who consult our center for parotid swelling. Radiological examinations were in favor of intraparotid cystic lesions. Both patients benefited from an excision whose histopathological study was in favor of primary parotid tuberculosis. The subsequent evolution was favorable under antituberculous treatment.

## Introduction

Tuberculosis is a chronic granulomatous infection caused by Mycobacterium tuberculosis or bovis that can affect all organs. Still a public health problem in Morocco, lung localization remains the main problem. At the level of the ENT sphere, lymph node involvement is the most frequent. The isolated location of tuberculosis in the salivary glands, especially the parotid gland, is extremely rare. There is no clinical, radiological or biological evidence to guide the diagnosis [1].

## Patient and observation

**Figure 1:**
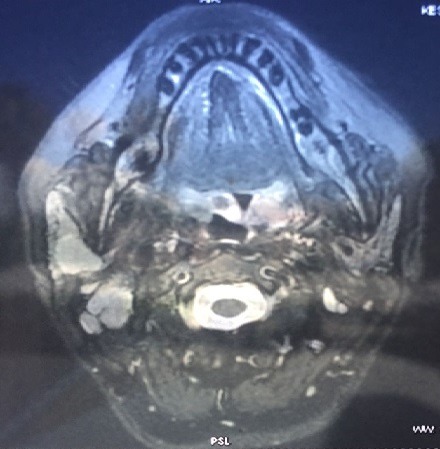
T1 axial section MRI after gadolinium injection

**Figure 2:**
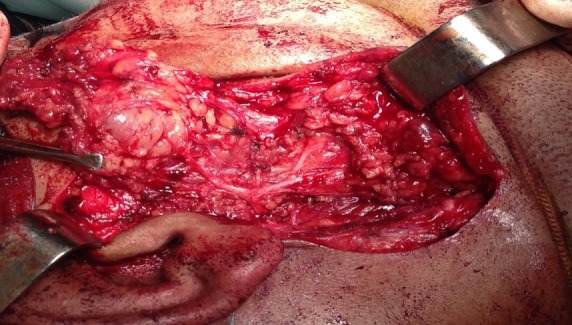
Exofacial parotidectomy with facial nerve preservation

**Figure 3:**
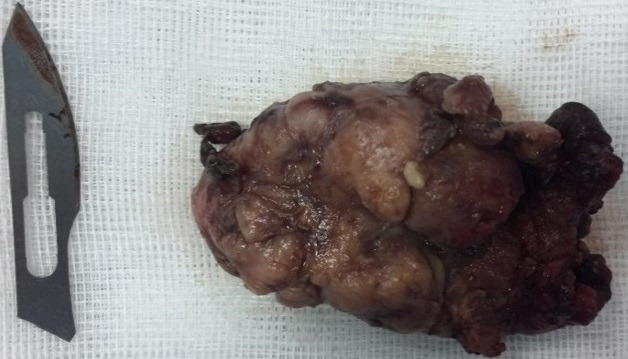
The resected mass

**Observation 1**: this was a 44-year-old woman, followed by iron deficiency anemia under iron supplementation, who developed an isolated swelling of the right parotid region, evolving for 5 months. Clinical examination: The patient was in good general condition, apyretic. The tumefaction was pretragal and measured 3cm of long axis, firm, well limited, mobile and painless, associated with multiple, hard and fixed homolateral jugulodigastric adenopathies. The controlateral parotid was with no particularities. There was no chewing discomfort, no trismus or endobuccal pus exit, no peripheral facial paralysis, and the upper aerodigestive tract was free. At Parotid MRI (Figure 1), the lesions straddled between the superficial and deep lobe of the right parotid, oval 20/15mm. It shows a nodule with low intensity T1 signal, and increased T2 signal, enhanced after Gadolinium administration, with presence of multiple homolateral, right jugulodigastric adenopathies. Its characteristics were suspicious of malignancy. The rest of the biological evaluation was normal. Chest x-rays showed no evidence of a possible chest location.The diagnosis of parotid cancer has been strongly suspected. To obtain histological evidence, we decided to perform a partial exofacial parotidectomy with dissection and preservation of the facial nerve (Figure 2). Anatomopathological examination of the resected mass (Figure 3) concluded a primary parotid and cervical lymph node tuberculosis. This was confirmed by definitive histological examination. The patient was put on anti-bacillary treatment (2RHZ/4RH) for six months, with significant improvement 6 months after the end of treatment.

**Figure 4:**
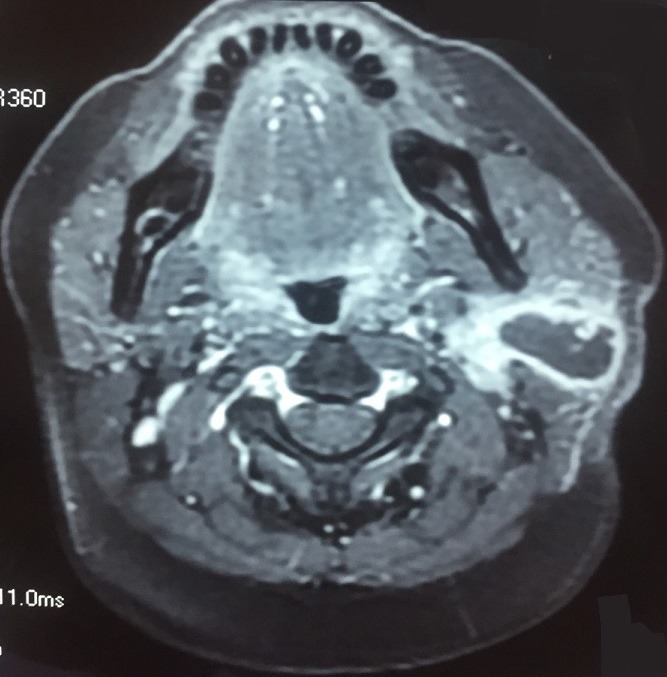
T1 axial section MRI after gadolinium injection

**Observation 2**: a 45-year-old woman, with no notable pathological history, consulted in our facility for a left sub-angulo-mandibular mass evolving for approximately 4 months. There were slight inflammatory signs nearby, particularly a slight redness with pain and fever at 38°C without weight loss, chewing discomfort, trismus, or pus exit at salivary orifices. The young woman was in good general condition and had a swelling of the left parotid area, firm, painless, measuring about 4 cm in long axis, and fixed. There was no limitation of the mouth opening or peripheral facial paralysis. Examination of the oral cavity and the opening of the stenon canal did not reveal any inflammation or pus output. Examination of the cervical lymph node areas was normal. The rest of the ENT and general examination was without any particularity. A cervical ultrasound was done to show a tumor lesion, measuring 29 x 28 x 14 mm, of mixed echostructure with central liquid predominance at the lower pole of the left parotid. Parotid MRI showed a lesional process in the deep compartment of the left parotid gland, with low intensity T1 signal, and in the other hand, high intensity signal in diffusion sequence with peripheral enhancement, measuring 30 x 29 x 15 mm. The right parotid and submandibular glands, in addition to the thyroid are without signal or morphological abnormalities. This was in favor of a left parotid abscess (Figure 4). The rest of the biological test, as well as the chest x-ray, were normal. The patient was operated benefiting conservative parotidectomy of the facial nerve. Histopathological study of the cyst wall found epithelial-gigantocellular granuloma with caseous necrosis. The culture of the puncture fluid on Lowenstein-Jensen medium revealed an acid-resistant bacillus, which allowed us to confirm the diagnosis of primary tuberculosis of the parotid gland. The young woman received a medical treatment of antituberculosis drugs combining an initial phase of 2 months based on rifampicin, isoniazid, ethambutol, pyrazinamide, followed by a consolidation phase of 4 months combining Rifampicin and Isoniazid. The evolution was favourable within 9 months after the end of the treatment.

## Discussion

Parotid tuberculosis is a rare entity of extrapulmonary tuberculosis and particularly of extranodal ENT localization. After Robert Koch had discovered the bacillus responsible in 1882, the first case of secondary parotid tuberculosis was reported in 1894 by De Paoli, and Von Stubenrauch described a second case in 1893, which is of primary disease. Since then to the present day the literature has reported 220 cases. It is a pathology that can reach all ages. But often, the young adult between 20 and 40 years, remains more incriminated. The child is rarely affected. Both sexes, male and female, are equally affected. Ninety percent of published cases come from the African and Asian continents, countries where tuberculosis is a public health problem. Parotid localization poses a real differential diagnostic challenge for practitioners because of the non-specific clinical picture, and because many paraclinical examinations are non-conclusive [1]. All the factors that contribute to the occurrence of tuberculosis in all its forms are also involved in parotid disease, namely low socio-economic levels, promiscuity, tuberculosis infection and immunosuppressive conditions such as HIV, diabetes and malnutrition. The consumption of unpasteurized raw milk also seems to be a cause since it is a source of contamination by bacillus M. Bovis. The common clinical presentation is a gradual increase in gland size over a period of 2 to 6 months on average, painless. Nevertheless, the duration of evolution is variable and can reach several years. This swelling looks firm and hard, sometimes nodular, with varying degrees of fixation in relation to the superficial and deep planes, giving a pseudo tumoral aspect. The location is often unilateral, rarely occurring in both parotid glands at the same time [2]. A fistulization of the swelling can direct the doctor towards the tubercular pathology, but generally the cutaneous plane remains intact with slight inflammatory signs. Trismus is rare in tubercular sialadenitis. Its presence, particularly with peripheral facial paralysis, raises doubts about a malignant tumor of the parotid gland. The parotid swelling can occur in isolation, as it can be associated with cervical adenopathy, and can direct the practitioner to malignant tumor pathology, as part of a locoregional extension of a parotid tumor. In primary disease, signs of tuberculis with night sweats, weightloss, asthenia and fever are rarely present [3]. The cervical parotid ultrasound test is simple to perform, the reason why it is performed in first intention in front of parotid swelling. The general appearance is characterized by an increase in the size of the gland, with one or more hypoechoic lesions of variable size, with clear limits, without posterior reinforcement. Cold parotid tubercular abscesses are badly limited hypoechoic or anechoic collection, with posterior reinforcement, sometimes containing intense echoes corresponding to debris. However, these aspects can be seen in other pathologies such as pleomorphic adenoma, carcinomas, lymphomas, fungal and parasitic infections, pyogenic abscesses or intraparotid adenopathies [4].The cervico-facial CT allows exploring the parotid gland in all its dimensions with great sensitivity compared to ultrasound. The tomodensitometry sections are made before and after injection of an iodized contrast medium, 5mm thick. However, the radiological aspects found are not specific and do not correlate lesions to parotid tuberculosis. Comparing our two cases and the results of the literature, it seems that the most frequent aspect is the presence of a thick-walled lesion, strongly contrasting with necrosis in the center, which is pathognomonic of tuberculosis. Nevertheless, recent studies have concluded that all aspects can be seen: homogeneous enhancement of the parotid gland; homogeneous enhancement with a non-enhanced eccentric micro cyst; contrasting thick-walled hypodense lesion; isolated or confluent hypodense nodular lesions. However, CT is still limited [5].

The cervico-facial MRI allows a good analysis of the parotid lodge and asserts the parotid nature of the lesion. It is considered superior to CT in the detection and analysis of a parotid tumor process. In the normal state, the parotid parenchyma shows a T1 signal more intense than the muscle but less than the peripheral fat. The glandular capsule appears fine and regular. In parotid tuberculosis, the lesion shows a low T1 signal, and high T2 signal, which can be delimited by a shell enhanced after injection of Gadolinium. However, this aspect is not specific. Other very frequent benign parotid tumors pose a problem of differential diagnosis, such as pleomorphic adenoma, which manifests itself by a low T1 signal and high T2 signal, always enhanced but sometimes heterogeneously [5,6]. A chest X-ray should be requested systematically in search of a possible primitive site. Anatomopathological examination is the gold standard in the diagnosis of parotid tuberculosis. Samples for this study were obtained from the cytopunction, biopsy curettage of the fistulous opening, open biopsy or parotidectomy procedure. The typical aspect is the presence of giganto-cellular epitheloid granulomas with caseous necrosis which is specific to tuberculosis and distinguishes it from other granulomatous diseases, such as sarcoidosis, where it is absent [7]. The differential diagnosis of parotid tuberculosis is mainly made with tumor pathology, other infectious parotiditis, systemic diseases and lithiasis. Previously, parotidectomy followed by bacillary treatment was the treatment of choice. Authors attributed a crucial role to parotidectomy in the successful dissemination of anti-tuberculosis drugs. Currently, all authors agree on the uselessness of parotidectomy and the effectiveness of medical treatment alone. From our two cases, we concluded that radiological examinations, namely ultrasound, CT and MRI and biological examinations, do not confer specific aspects and do not allow the diagnosis to be made, explaining our use of an exofacial parotidectomy for diagnostic and therapeutic purposes [8]. Anti-tuberculosis treatment in Morocco is part of tuberculosis control program, recommended by the Ministry of Public Health. It is a free standardised treatment, controlled and administered mainly on an outpatient basis in public health centers; hospitalization is the prerogative of complicated or serious forms. Clinical forms of tuberculosis are classified into four categories according to the therapeutic priorities of the National Tuberculosis Program; the category I includes new cases of TPM+ (pulmonary tuberculosis with positive microscopy) and severe forms. Category II combines treatment failure and relapse. Category III includes TPM0 (pulmonary tuberculosis with negative microscopy), TPM0C+ (pulmonary tuberculosis with negative microscopy and Positive culture), PI (primary infection) and PET (extrapulmonary tuberculosis). Category IV includes chronic and multidrug-resistant cases. Parotid tuberculosis falls into the category III, and therefore the medication plan adopted is 2RHEZ/4RH. It combines an initial 2-month phase based on 4 so-called first-line antibacterial molecules (RHEZ): rifampicin; isoniazid; ethambutol, pyrazinamide; followed by a 4-month consolidation phase combining (RH) Rifampicin and Isoniazid [8,9]. Direct supervision of medication intake is performed during the initial phase of treatment, with bio clinical and/or radiological monitoring of efficiency and tolerance to antibacterial agents throughout the duration of therapeutic compliance. The risk of relapse or non-healing, despite well managed treatment, is 1%. These failures are due to the appearance of resistant strains of BK to antibacterial agents [9].

## Conclusion

Tuberculosis of the parotid gland is rare and can occur in different clinical forms. Often confused with neoplastic pathology. Since Morocco is a country where tuberculosis is a major public health problem, the ENT surgeon must evoke this diagnosis before any parotid swelling, and this requires knowledge of the epidemiological, clinical and paraclinical aspects allowing an early positive diagnosis before arriving at surgery. Since the antibacterial treatment alone is effective allowing the regression of the parotid swelling and the elimination of the tubercular focus; the use of parotidectomy for diagnostic and/or therapeutic purposes is no longer a hot topic.

## Competing interests

The authors declare no competing interests.
